# Supplementary material for: Patterns of Intron Gain and Loss in Fungi
Source: PLoS Biol. 2004 Nov 30;2(12):e422. doi: 10.1371/journal.pbio.0020422 (PMC532390; doi:10.1371/journal.pbio.0020422)
Supplement: Table S1 — Also available at http://genes.mit.edu/NielsenEtAl/. (4.3 MB ZIP). [file pbio.0020422.st001.zip › NielsenEtAl/html/349.html]

AN1543.1.NCU02580.1.MG03619.1.FG09341.1


```
 CLUSTAL W (1.82) Multiple Sequence Alignments - Introns Inserted


Sequence 1: MG03619.1	619 aa
Sequence 2: FG09341.1	630 aa
Sequence 3: NCU02580.1	616 aa
Sequence 4: AN1543.1	627 aa
Alignment Length: 633 aa
Number Identitical Residues: 444 aa
Alignment Score (without introns) 19321


MG03619.1 	MPSRVIVVGGGL1SGLSAAHTIYLAGGNVVVLDKQG1FFGGNSTKATSGINGALTRTQVE
NCU02580.1	MAPRVIVVGGGL1SGLSAAHTIYLAGGNVVVLDKQG~FFGGNSTKATSGINGALTRTQVE
FG09341.1 	MAPRVIVVGGGL1SGLSAAHTIYLAGGNVVVLDKQG1FFGGNSTKATSGINGALTRTQVD
AN1543.1  	MAPRVIVVGGGL1SGLSAAHTVYLNGGSVLVLDKQA1FFGGNSTKATSGINGALTRTQVD
          	*..********* ********:** **.*:*****. **********************:

MG03619.1 	HGIQDSVKQFYDDTLKSARDKARPDLIKVLTYKSAAAVEWLQDVFNLDLTLVSRLG2GHS
NCU02580.1	LGIQDSVKQFYDDTLKSARDKARPDLIKVLTYKSAAAVEWLQDVFKLDLTLVSRLG2GHS
FG09341.1 	TGIPDSVKQFYDDTLKSARDKARPDLIKVLTYKSAAAVEWLQDEFNLDLTLVSRLG2GHS
AN1543.1  	LNIRDSVKQFYDDTLKSARDKARPDLIKVLTYKSAAAVEWLQDVFNLDLTLVSRLG2GHS
          	 .* *************************************** *:********** ***

MG03619.1 	QPRTHRGHDAKFPGMAITYALMQRLEELAETEPHRVEIVKKARVTNLNKEGNKVTGVTYE
NCU02580.1	QPRTHRGHDAKFPGMAITYALMQRLEELAETEPERVQIIKKARVTSLNKEGNKVTGVKYE
FG09341.1 	QPRTHRGHDAKFPGMAITYALMQRLEELAESEPGRVEIVKKARVTELNKEGNKVTGVKYE
AN1543.1  	QPRTHRGHDAKFPGMAITYALMQRLEELSESDPDRVQIIKKARVTAVNKDGNAVTGVTFE
          	****************************:*::* **:*:****** :**:** ****.:*

MG03619.1 	HDGQKHTMDGPVVLATGGYAADFSETSLLKKHRPDTYGLATTNGSHATGDGQKMVMAIGG
NCU02580.1	VNGETQSLDGPVVLATGGYAADFTEDSLLKKHRPDVYGLATTNGTHATGDGQKMVMAIGG
FG09341.1 	HNGEIVSIDGPVVLATGGYAADFSDSSLLKKHRPDTYGLATTNGTHATGDGQKMVMAIGG
AN1543.1  	VDGETKTADGVVILATGGYAADFGDSSLLKKHRPDTFGLSSTNGTHATGDGQKMLMSIGA
          	 :*:  : ** *:********** : *********.:**::***:*********:*:**.

MG03619.1 	NGIDMDKVQVHPTGLVDPKDPGSKWKFLAAEALRGEGGLLLNADGDRFCDELGHRDYVSG
NCU02580.1	NGIDMDKVQVHPTGLVDPKDPNSKWKFLAAEALRGEGGILLNGDGDRFCDELGHRDYVSG
FG09341.1 	NGIDMDKVQVHPTGLVDPKDPGSKWKFLAAEALRGEGGLLLNADGDRFCDELGHRDYVSG
AN1543.1  	NGIDMDKVQVHPTGLVDPKDPTAKFKFLAAEALRGEGGLLLNSDGERFSDELGHRDYVSG
          	********************* :*:*************:***.**:**.***********

MG03619.1 	MMWKEKDKGKFPIRLILNSKASNTLDFHTRHYSGRGLMKKMTGQQLAKEIGCTPEHLQKT
NCU02580.1	MIHKEKDKGKYPVRLVLNSKASKTLDFHTRHYSGRGLMKKMTGQELAKEIGCTPEHLQKT
FG09341.1 	MMWEEKKKNKFPIRLILNSKASKVLDFHTRHYSGRGLMKKMTGKELAKEIGCTPEHLQKT
AN1543.1  	QMWKEKEKGKWPIRLVLNSKASKVLDFHTRHYSGRGLMKKMTGKELAKEIGCGEAKLQKT
          	 : :**.*.*:*:**:******:.*******************::*******   :****

MG03619.1 	FTTYNAIAEGKQKDPWGKKFFHNLPLDINDDFHVSLMEPVLHFTMGGVEINDKSEVLGQD
NCU02580.1	FQTYNAIAEGKQKDPWGKKFFHNLPVDINDDFHVSIMEPVLHFTMGGIEINDKAQVLNSE
FG09341.1 	FSTYNDIADGKQKDPWGKKFFHNLPVNVDDDFHVAVMEPVLHFTMGGIEINDKAQVLNQE
AN1543.1  	FEDYNAIAEGKKKDPWNKRFFHNLPFSIDDEFHVALMEPVLHFTMGGIEINDRAEVLNSE
          	*  ** **:**:****.*:******..::*:***::***********:****:::**..:

MG03619.1 	KKPIEGLYACGELAGGVHGANRLGGSSLLGCVVYGRVAGDTASNYLFRQALSGGAG-AAQ
NCU02580.1	QKPFEGLYACGELAGGVHGANRLGGSSLLGCVVYGRVAGDSASNYLFQEALKGNAG--AA
FG09341.1 	KQPFEGLYACGELAGGVHGANRLGGSSLLGCVVYGRVAGDTASNYLFQNALKGGAGSAAE
AN1543.1  	HKPFDGLYACGELAGGVHGANRLGGSSLLGCVVYGRVAGDSASEYLFKKLVAGGGSSAQQ
          	::*::***********************************:**:***:: : *...::  

MG03619.1 	RLGQISLHIDPTQPGKVSVEWG-----SGAGAAA-----ASTQSGAGNVAATQ-AESAKP
NCU02580.1	RLGQIALHIDPSQPGKISVQWG-----GESGAQA-------TANTEAPKKDTA-AAAPKK
FG09341.1 	RVGQISLHLDPSIPNQVTVSWGAPGAASGSGAPSKVDEHASVAAGPNPVKEDG-AKAAKP
AN1543.1  	RLGQISLHIDPATPGKVSVEWN------GAGAAKASGTNPAVSTAPLPANADAPAETSKP
          	*:***:**:**: *.:::*.*.       :**    .  .:.           .* :.* 

MG03619.1 	--NNPKEFKIPEKEFTMEEVAKHNKKDDLWVVVKGVVMDLSDWLEEHPGGPQAIMNFMGR
NCU02580.1	--ENPKAFTIPDKEYTMEEVAKHNKKEDLWVVVKGVVLDLTNWLEEHPGGVQALLNFMGR
FG09341.1 	--NDPKAFKVPEKEYTMEEIAEHNTKDNVWVVVKGVVLDLSDWLEEHPGGVQAILNFMGR
AN1543.1  	PTKAPKKFTIPETEYSMEEVAKHNKKDDLWIVVKGIILDVTNWLDEHPGGANALFNFMGR
          	.:: ** *.:*:.*::***:*:**.*:::*:****:::*:::**:***** :*::*****

MG03619.1 	DATEE~FEMLHDDEVIPKYAPSQVIGRVKGVKPTLEI
NCU02580.1	DATEE~FEMLHDDEVIPKYAPEQVIGRVKGQKVTLEV
FG09341.1 	DATEE~FEMLHDDEVIPKYAPQQVIGRVKGQEVTLEP
AN1543.1  	DATEE1FEMLHDDEVIPKYAPHIVIGRVKGQTPSLEY
          	***** ***************  *******   :**
```
